# Proteomic and Isotopic Response of *Desulfovibrio vulgaris* to DsrC Perturbation

**DOI:** 10.3389/fmicb.2019.00658

**Published:** 2019-04-11

**Authors:** William D. Leavitt, Sofia S. Venceslau, Jacob Waldbauer, Derek A. Smith, Inês A. Cardoso Pereira, Alexander S. Bradley

**Affiliations:** ^1^Department of Earth Sciences, Dartmouth College, Hanover, NH, United States; ^2^Department of Earth and Planetary Sciences, Washington University in St. Louis, St. Louis, MO, United States; ^3^Department of Biological Sciences, Dartmouth College, Hanover, NH, United States; ^4^Department of Chemistry, Dartmouth College, Hanover, NH, United States; ^5^Instituto de Tecnologia Química e Biológica António Xavier, Universidade Nova de Lisboa, Oeiras, Portugal; ^6^Department of the Geophysical Sciences, University of Chicago, Chicago, IL, United States; ^7^Division of Biology and Biomedical Sciences, Washington University in St. Louis, St. Louis, MO, United States

**Keywords:** proteomics, chemostat, microbial sulfate reduction, sulfur isotope fractionation, microbial energy metabolism

## Abstract

Dissimilatory sulfate reduction is a microbial energy metabolism that can produce sulfur isotopic fractionations over a large range in magnitude. Calibrating sulfur isotopic fractionation in laboratory experiments allows for better interpretations of sulfur isotopes in modern sediments and ancient sedimentary rocks. The proteins involved in sulfate reduction are expressed in response to environmental conditions, and are collectively responsible for the net isotopic fractionation between sulfate and sulfide. We examined the role of DsrC, a key component of the sulfate reduction pathway, by comparing wildtype *Desulfovibrio vulgaris* DSM 644^T^ to strain IPFG07, a mutant deficient in DsrC production. Both strains were cultivated in parallel chemostat reactors at identical turnover times and cell specific sulfate reduction rates. Under these conditions, sulfur isotopic fractionations between sulfate and sulfide of 17.3 ± 0.5‰ or 12.6 ± 0.5‰ were recorded for the wildtype or mutant, respectively. The enzymatic machinery that produced these different fractionations was revealed by quantitative proteomics. Results are consistent with a cellular-level response that throttled the supply of electrons and sulfur supply through the sulfate reduction pathway more in the mutant relative to the wildtype, independent of rate. We conclude that the smaller fractionation observed in the mutant strain is a consequence of sulfate reduction that proceeded at a rate that consumed a greater proportion of the strains overall capacity for sulfate reduction. These observations have consequences for models of sulfate reducer metabolism and how it yields different isotopic fractionations, notably, the role of DsrC in central energy metabolism.

## Introduction

Sulfate (SO_4_^2−^) reduction coupled to organic matter oxidation is a common microbial energy metabolism. Microbial sulfate reduction (MSR) is performed by members of the archaeal and bacterial domains (Rabus et al., 2015), and the flux of matter and energy through MSR may account for up to half the organic carbon remineralized in marine sediments (Jørgensen, 1982; Bowles et al., 2014). As a key link between Earths S and C cycles, MSR has been operational for more than three billion years (Holland, 1973; Garrels and Lerman, 1981; Fike et al., 2015) and today the burial of sulfides derived from MSR balances at least one-fifth of the oxygen that has accumulated in the atmosphere (Hayes and Waldbauer, 2006). Sulfate reducing microorganisms also perform cryptic yet key roles in the biogeochemical cycles of other elements such as nitrogen, oxygen, and iron in lakes, bogs, oceans, soils, sediments and oil reservoirs (c.f. Canfield et al., 2010; Holmkvist et al., 2011; Flynn et al., 2014; Müller et al., 2014; Hansel et al., 2015; Wasmund et al., 2017; Anantharaman et al., 2018).

Microbial sulfate reducers can leave a record of their past activity in the form of geologically robust compounds. Sulfur-bearing minerals, such as sulfides, sulfates and geostable organic matter can accumulate in sedimentary rocks, some of which are stable for up to billions of years (Holland, 1973; Claypool et al., 1980; Bontognali et al., 2012). Sulfur (S) isotopic ratios within these compounds can be better understood through experimental approaches (Thode et al., 1951; Chambers et al., 1975; Sim et al., 2011a; Leavitt et al., 2013). The ultimate goal of such works is to understand how the MSR pathway and environmental conditions interacted in the past to generate the S isotopic signatures preserved for millions of years.

From the modern experimental literature two clear relationships have emerged. First, during MSR the magnitude of sulfur isotope fractionation between sulfate and sulfide increases in response to increased sulfate concentration, and can be modeled in a manner similar to a Michaelis-Menten-Monod-type relationship (Habicht et al., 2005; Bradley et al., 2016). Second, the magnitude of fractionation decreases linearly as cell-specific sulfate reduction rate increases logarithmically (Chambers et al., 1975; Sim et al., 2011a; Leavitt et al., 2013). Recent modeling studies have attempted to describe these relationships under all environmental conditions and as a consequence of the inherent enzymatic steps, each of which may express an isotope fractionation (Bradley et al., 2011; Wing and Halevy, 2014; Bradley et al., 2016; Wenk et al., 2017). A few experimental studies have examined the potential role of intermediate S species on fractionation on net S isotopic fractionation (Smock et al., 1998; Leavitt et al., 2014; Bertran et al., 2018b), as have some model studies (Rees, 1973; Brunner and Bernasconi, 2005; Farquhar et al., 2007; Johnston et al., 2007; Turchyn et al., 2010; Brunner et al., 2012; Bertran et al., 2018a). Other studies have examined fractionation imposed by the individual enzymes, such as DsrAB (Leavitt et al., 2015) or AprAB (Sim et al., 2019) or highlighted the importance of material fluxes of sulfur through the most up-to-date MSR metabolic network (Bradley et al., 2016).

Different S isotopic fractionations are expressed by closely related strains when cultured under identical experimental conditions (Bradley et al., 2016). This suggests widespread natural variation in the strain-specific flux of material through the same biochemical network, and/or differences in the enzyme-specific fractionations of the different strains. This report examines the role of a single protein in the MSR reaction network, the highly expressed and requisite sulfur carrier DsrC.

**FIGURE 1:**
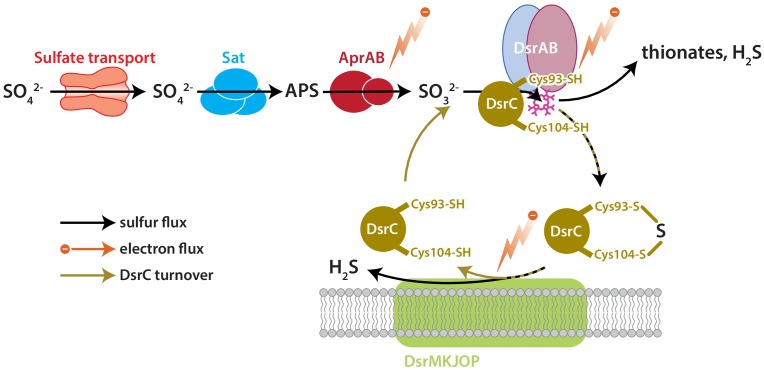
The key enzyme-catalyzed reactions of MSR. Sulfate is imported to the cell through transporters, activated to APS by Sat, and reduced to sulfite by AprAB. Sulfite is reduced to H_2_S by DsrAB at the siroheme active site (pink), which partially reduces sulfite to zero-valent S. Approximately zero-valent S is carried to the membrane by DsrC, where it undergoes final reduction to H_2_S (Santos et al., 2015). There is potential for a branching flux of sulfur at DsrAB, which can produce ancillary H_2_S by complete reduction of sulfite, or produce thionates by the partial reduction of sulfite and scavenging of that pool by intracellular sulfite (Leavitt et al., 2015). The amount of branching flux depends on the rate of electron supply, sulfur supply, and reduced-DsrC supply. Key steps predicted to affect fraction involve the reductive steps at AprAB, DsrAB, and DsrC.

DsrC is the key sulfur transfer protein in the terminal step of dissimilatory sulfate reduction (Santos et al., 2015). DsrC directly interacts with DsrAB to carry a partially reduced sulfur atom from the cytoplasmic DsrAB complex to the membrane, where the DsrC-bound S accepts electrons from the DsrMKJOP complex, is reduced, and thus released as H_2_S (Venceslau et al., 2014; Santos et al., 2015). DsrAB has been the focus of S isotopic fractionation study because sulfur bound to this enzyme has multiple fates: reduction to sulfide through DsrC, reduction to sulfide directly, or loss as thionates (S_x_O_y_^2−^) (Leavitt et al., 2015). The fate(s) of S and rate(s) of reaction(s) relate to the availability of S-bearing molecules, reducing equivalents (electrons), and DsrC, all as reactants to the catalyst DsrAB (Figure 1). Reductant availability or sulfite (SO_3_^2−^) availability can be perturbed through environmental concentrations of electron donors or sulfate. In this study we examine the effects of modulating DsrC availability. Our recent study also examined this question, but the results were confounded by differences in growth rates among the strains due to the experiments being closed-system batch in design (Leavitt et al., 2016).

In this study, we examined the role of DsrC in setting the observed S isotope fractionation between sulfate and sulfide during MSR. We utilized a previously constructed mutant strain of *Desulfovibrio vulgaris* in which DsrC expression has been significantly reduced relative to the wildtype (Santos et al., 2015). Both wildtype and mutant were grown in parallel continuous cultures at the same dilution rate. For each strain we examined the steady-state net S isotopic fractionation and proteome content. We emphasize that most omics studies compare a complete deletion strain to the wildtype or background strain, but this is not the case here. To-date no group has been able to knock out *dsrC* and generate viable strains (a fatal mutation). Due to the impossibility of generating a *dsrC* deletion strain, we compare the proteome of two strains that produce quantifiably different amounts of DsrC (Santos et al., 2015). Our approach provides insight into how differences in cellular physiology among strains with near identical genetic backgrounds, and independent of environmental conditions, can result in different sulfur isotopic fractionations.

## Materials and Methods

### Strains and Cultivation

The *dsrC*-mutant strain IPFG07 derived from the parent *D. vulgaris* DSM 644^T^ wildtype (WT) was described previously (Santos et al., 2015). Batch culture medium and cultivation conditions were reproduced from Leavitt et al. (2016), with the following modifications: media contained 30 mM lactate as the electron donor and 30 mM sulfate as the electron acceptor; under these conditions, 50% of the sulfate was consumed. All chemostat experiments were conducted at 30.5 ± 0.1°C and pH 7.2 ± 0.1, with all batch experiments performed at 30°C with initial pH 7.2. Prior work was performed at 37°C (Leavitt et al., 2016).

### Chemostat (Open-System) Setup, Operation, Sampling, and Calculations

Two open-system chemostats were operated in parallel, one for cultivation of the WT strain and one for strain IPFG07. Each chemostat was held at a constant steady-state dilution rate of 1.1 per day (Novick and Szilard, 1950). The design of the chemostats followed that of Leavitt et al. (2013), but with smaller working volumes (1 L instead of 3 L). In the reactors, all surfaces in contact with sulfide (gas or liquid) were glass, PEEK (“polyether ether ketone”), or PTFE (“polytetrafluoroethylene”) to avoid re-oxidation of the biogenic sulfide. Prior to each chemostat run, the 1 L reactor vessel (BellcoGlass, part no. 1964-06660) was filled with 0.3 L of growth medium. The medium was then sterilized in an autoclave, degassed with high purity O_2_-free N_2_, and the pH adjusted to 7.2 ± 0.1 via a titration pump (Etatron, DLX pH-RX/MBB metering pump) which supplied a degassed and sterilized titrant, either 1 M HCl or 1 M NaOH.

Prior to inoculation in the chemostats, each strain was cultivated in batch through at least three transfers, to acclimate the strains to the growth medium. During batch growth, the specific growth-rate (μ) for this medium was estimated for each strain (Dataframe 01, Supplementary Figure S1). Following these preparations, 100 mL of each strain was harvested at mid-exponential phase and inoculated into a chemostat vessel. Each inoculated reactor was initially operated only with gas flux (i.e., as a closed system to liquid flux, but open to gas flux) until the culture had reached late log-phase. At this point, inflow and outflow pumps (Ismatec SC0816 with 2-stop Tygon tubing with a 0.64 mm internal diameter) were activated. In order to avoid wash-out, chemostat dilution rates were set slower than the batch-determined maximum growth rates (μ_batch_) of the two strains (Herbert et al., 1956). Sampling of the effluent liquid (L) and gas (G), the reservoir medium (M), and intra-reactor liquid (R), was performed approximately each turnover (once a day). These samples were used to quantify sulfur and carbon species during each sampling interval, and to measure the major isotope compositions of sulfate and sulfide (see section “Analytical Procedures”). Sampling was more frequent prior to the reactors achieving steady-state. Specific growth rate was calculated by measuring the dilution rate and the rate of change in optical density between time-points, as previously reported (Leavitt et al., 2013; Bradley et al., 2016). Prior to sampling for isotopes or proteomics, we required that populations must be growing within 10% of steady state (D = μ), as determined by the volumes collected per interval and the optical density readings.

Sampling procedures, analytical measurements and calculations follow those used in recent studies (Leavitt et al., 2013, 2016; Bradley et al., 2016). We tracked the concentrations and fluxes of sulfate, sulfide, lactate, and acetate, and measured optical density. Measurements of the following biological and geochemical parameters recorded on a minimum of five time-points per reactor: temperature, pH, δ^34^S of effluent sulfate and sulfide, and the proteomic content of MSR populations. More frequent sampling was performed for some measurements (see Dataframes). We calculated the fractionation between sulfate and sulfide (^34^ε) from the measured δ^34^S values of effluent sulfate and sulfide. Variability in ^34^S/^32^S of a measured pool, y, is reported as δ^34^S_y_: δ^34^S_y_ = [(^34^S/^32^S)_sample_]/[(^34^S/^32^S)_standard_ − 1] × 1000, where y is a distinct S-bearing species or operationally defined pool. The difference between two pools (y = A or B, e.g., sulfate and sulfide) is calculated as: ^34^ε_A−B_ = (^34^α_A−B_ − 1) × 1000, where ^34^α_A−B_ = [(^34^S/^32^S)_A_/(^34^S/^32^S)_B_].

### Analytical Procedures

Optical density measurements were made on a UV/visible spectrophotometer (NanoDrop; Thermo Fisher Scientific) by comparing duplicate 0.7 mL to a blank (deionized water). Effluent liquid or intra-reactor samples for sulfate, lactate, and acetate concentration measurements were centrifuged at 18,000 × *g* for 10 min to remove ZnS and cell solids, and then ion concentrations were determined by suppressed anion chromatography with conductivity detection (Dionex ICS-2000, AS11 column) (Sim et al., 2017). Sulfide concentrations were measured by a modified methylene blue “Cline” method in duplicate (SD ± 6%; detection limit 5 μM) as described previously (Leavitt et al., 2016).

Samples for sulfur isotope analysis (δ^34^S) were prepared by precipitating sulfate as BaSO_4_ (from L and M samples) and sulfide as Ag_2_S (from G samples), following the preparative schemes described previously (Leavitt et al., 2013, 2014, 2016; Bradley et al., 2016). For S isotope analysis, samples were converted to SO_2_ at 1040°C in the presence of excess V_2_O_5_ in an elemental analyzer (Costech ECS 4010), coupled inline to an isotope ratio monitoring mass spectrometer (Thermo-Finnegan DELTA V Plus). Each sample was measured at least twice, and precision is estimated at ±0.3‰ (1σ).

### Proteomics Analysis Methods

Cell material for proteomic analysis were collected from five separate chemostat turnovers for each strain. These turnovers are denoted as tr1, tr2, …, tr5 (turnovers 1–5). Each reactor achieved steady-state for a minimum of three cumulative reactor turnovers prior to sampling for stable S isotope and proteome measurements, and samples were only collected at the appropriate dilution rates. At each turnover sampled, 50 mL of reactor effluent was collected anoxically and aseptically, then centrifuged at 4°C for 10 min at 5000 × *g*. The supernatant was discarded and the cell pellet was flash-frozen in liquid nitrogen, then stored at –80°C until protein extraction and analysis.

Cell pellets were extracted by heating (95°C, 20 min) and vortexed in a reducing and denaturing SDS (1%)/Tris (200 mM, pH 8.0)/DTT (10 mM) buffer, and cysteine thiols alkylated with 40 mM iodoacetamide. Proteins were purified by a modified eFASP (enhanced filter-aided sample preparation) protocol (Erde et al., 2014), using Sartorius Vivacon 500 concentrators (30 kDa nominal cutoff). Proteins were digested with MS-grade trypsin (37°C, overnight), and peptides were eluted from the concentrator dried by vacuum centrifugation. For quantitative analysis, peptides were isotopically labeled at both N- and C-termini using the diDO-IPTL methodology (Waldbauer et al., 2017). Briefly, C-termini were labeled with either oxygen-16 or -18 by enzymatic exchange in isotopic water of >98 atom% enrichment. N-termini were labeled with either un- or dideuterated formaldehyde via reductive alkylation using sodium cyanoborohydride. Wildtype samples were labeled with H_2_^16^O and d2-formaldehyde and IPFG07 samples were labeled with H_2_^18^O and d0-formaldehyde; labeled peptides from the respective timepoints were mixed and analyzed by LC-MS for protein expression quantification.

For LC-MS analysis, peptide samples were separated on a capillary C18 column (Thermo Acclaim PepMap 100 Å, 2 μm particles, 50 μm I.D. × 50 cm length) using a water-acetonitrile + 0.1% formic acid gradient (2–50% AcN over 180 min) at 90 nL/min using a Dionex Ultimate 3000 LC system with nanoelectrospray ionization (Proxeon Nanospray Flex source). Mass spectra were collected on an Orbitrap Elite mass spectrometer (Thermo Fisher Scientific) operating in a data-dependent acquisition (DDA) mode, with one high-resolution (120,000 *m*/Δ*m*) MS1 parent ion full scan triggering Rapid-mode 15 MS2 CID fragment ion scans of selected precursors. Proteomic mass spectral data were analyzed using MorpheusFromAnotherPlace (MFAP; Waldbauer et al., 2017), using the predicted proteome of *D. vulgaris* Hildenborough as a search database. Precursor and product ion mass tolerances for MFAP searches were set to 20 ppm and 0.6 Da, respectively. Static cysteine carbamidomethylation and variable methionine oxidation, N-terminal (d4)-dimethylation, and C-terminal ^18^O_2_ were included as modifications. All proteomic mass spectral data have been deposited in the MassIVE repository under accession MSV000083165^1^, with processed output in the Supplementary Material. FigShare link in the Supplementary Material and associated DOI^2^.

False discovery rate (FDR) for peptide-spectrum matches was controlled by target-decoy searching to <0.5%. Protein-level relative abundances and standard errors were calculated in *R* using the Arm postprocessing scripts for diDO-IPTL data^3^, and *p*-values against the null hypothesis of equal abundance in WT and mutant were calculated at each timepoint by *t*-test. These *p*-values were combined across time points by Fisher’s method, and familywise FDR for differential protein abundance between WT and mutant controlled by the method of Benjamini and Hochberg (1995) (hereafter *BH*) (Benjamini and Hochberg, 1995). When examining the entire dataset, the significance cutoff was *p* < 0.01 (subsequent FDR at 0.0575), allowing the inclusion of DsrC. The FDR is relaxed from 0.05 to 0.0575 because DsrC is unequivocally less expressed in IPFG07 than in the WT, as was recently demonstrated via Western blot (Santos et al., 2015). When examining only the energy metabolism data, we are sub-setting from the whole-proteome and so enforce a stricter significance threshold as the number of proteins examined in a given subset influences the significance threshold [see derivation for the Benjamini and Hochberg (BH) significance threshold (Benjamini and Hochberg, 1995)]. For the energy metabolism subset, the FDR was set to a standard 0.05 for the BH test, with a corresponding *p*-value threshold of <0.02. Data is reported as log_2_-transformed weighted ratios of protein abundance in the IPFG07 strain relative to the WT.

Genome data for *D. vulgaris* DSM 644^T^ and other bacteria were obtained from MicrobesOnline^4^ and Integrated Microbial Genomes (IMG^5^). Information about Clusters of Orthologous Groups of proteins (COGs) were obtained from the MicrobesOnline database.

## Results

### Rates and Sulfur Isotopic Fractionations

**FIGURE 2:**
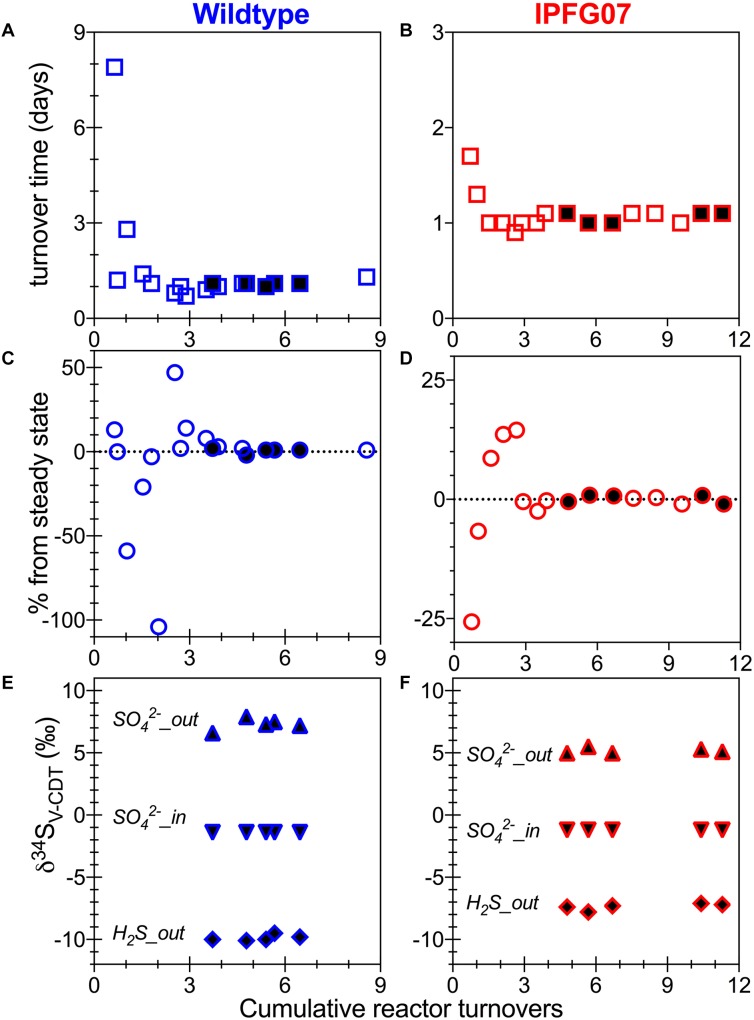
Chemostat timepoints: for WT **(A,C,E)** and IPFG07 mutant **(B,D,F)**. Panels **A** and **B** are calculated turnover times. Panels **C** and **D** are departures from steady-state. Panels **E** and **F** are sulfate and sulfide S isotopic compositions on the international scale (V-PDB). In all filled symbols are from the time points at which proteome samples were also collected.

Batch growth of each strain was performed prior to the chemostat experiments to determine the maximum specific growth-rate possible under these medium and temperature conditions. The wildtype grew almost five times faster than IPFG07 at 0.24 and 0.05 doublings per hour, respectively (Supplementary Figure S1). Quantitation of each chemostats turnover time, departure from steady state and the sulfur isotopic ratios at each of the five-time point are presented in Figure 2.

**FIGURE 3:**
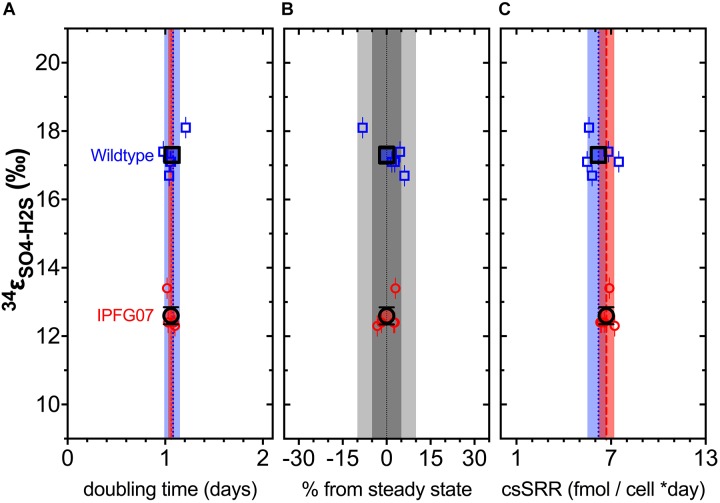
Chemostat samples: **(A)** Steady-state doubling time; **(B)** Departure from steady-state (dilution rate and growth rate); Panel **C** shows cell specific sulfate reduction rates, each plotted versus sulfur isotope fractionation. Individual values for WT (blue squares) and IPFG07 (red circles) reactors from each of the five separate turnover time points with the mean in bold. In panels **A** and **C,** the dashed lines are the mean and shaded areas are the CI_95%_ for WT (blue) and IPFG07 (red). In panel **B,** the dark and light gray areas indicate ±5% and ±10% departures from steady-state, respectively.

In the chemostats, the doubling time were statistically indistinguishable at 1.07 ± 0.08 vs. 1.06 ± 0.03 days for the WT and IPFG07, respectively (Figure 3A) during the five reactor turnover intervals sampled, as were optical densities (A_600_), 0.62 ± 0.06 and 0.60 ± 0.01, respectively (Dataframe 01). Deviation from steady-state (estimated as the difference between the observed dilution rate and growth rate) was +6 to –8% for the WT samples and at +3 to –3% for IPFG07 samples (Figure 3B). Both WT and IPFG07 reactor populations oxidized all lactate, coupled to sulfate reduction to sulfide at the canonical 2:1 stoichiometry, leaving behind 48 and 46% (±6%) of sulfate provided (see Dataframes on FigShare).

Reactor turnover times for the WT and IPFG07 were statistically indistinguishable during all of the five sampling intervals (Figure 3C). The cell-specific sulfate reduction rates (csSRR) were also identical, within analytical uncertainty: WT and the IPFG07 mutant: 6.2 ± 1.4 vs. 6.7 ± 1.0 fmol of sulfur per cell per day. Rates were calculated from both sulfate and sulfide data, were within error of each other, then averaged. The magnitude of sulfur isotopic fractionation between sulfate and sulfide differed between the strains (Figure 3). The net sulfur isotopic fractionation (^34^ε_H_2_S/SO_4__) was 17.3 ± 0.5‰ for WT vs. 12.6 ± 0.5‰ for IPFG07. All recorded data and calculations are available in the Supplementary Material and via the FigShare DOI^6^.

### Proteomic Profiles

**FIGURE 4:**
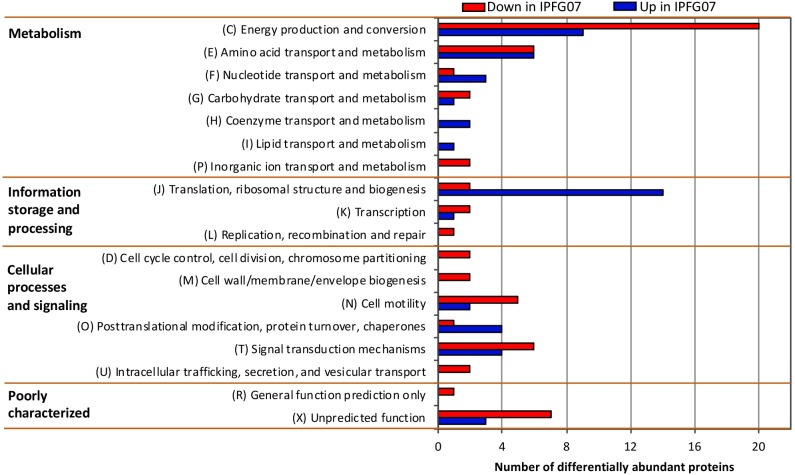
Clusters of orthologous groups (COGs) for the 99 proteins identified in all five biological replicates from both IPFG07 and WT samples that were differentially expressed (see section “Materials and Methods” for statistical significance calculations).

Proteomes from each of the five chemostat turnovers for WT and IPFG07 were compared quantitatively using the diDO-IPTL methodology (Waldbauer et al., 2017). The genome of *D. vulgaris* DSM 644^T^ has 3535 predicted protein-coding genes, and we detected a total of 1221 unique proteins, corresponding to 35% genome coverage. From these, 276/1221 proteins were quantified at all five time points from both strains. In Supplementary Figure S2 (Supplementary Figure S4, volcano plots) the differential abundance for each protein from each strain at all sampling points, plotted versus significance of detection (Supplementary Figures S2, S4). These 276 proteins constitute the set explored for differential expression between the WT and mutant strains. From these 276, 99 proteins were differentially expressed, with 55 under-expressed and 44 over-expressed in IPFG07 relative to WT (Supplementary Figures S2, S3). The proteins detected in all five replicates of both strains were then categorized by their clusters of orthologous groups (COGs) classification (Figure 4). The largest group of differentially expressed proteins belong to the Metabolism category, particularly proteins in Group C, e*nergy production and conservation* (∼30%). The most differentiated proteins were Group J, belonging to *translation, ribosomal structure, and biogenesis* category (16%), followed by Group E, *amino acid transport and metabolism* (12%), and finally Group X, *hypothetical proteins* (10%), and Group T *signal transduction mechanisms* (10%). Two protein classes are difficult to detect herein due to technical reasons: the integral membrane proteins and small cytochrome *c* proteins. The prior is insoluble in aqueous polar solvents and thus less likely to extract and derivatize without special considerations, the latter generate non-unique spectra. In *D. vulgaris* there are several proteins belonging to these groups, particularly membrane-bound (apolar) redox complexes, which would be of special interest here, so this is a limitation of the proteomic analysis. Nevertheless, both cases require a specialized extraction and/or analytical regime, and was beyond the goals of this study.

### Core Sulfate Reduction Proteins

The *Dv*H genome contains 210 proteins associated with energy metabolism COG. From this group, 59 were quantified in all five replicates in both strains, of which 29 were differentially expressed between strains (Supplementary Figure S3). Proteins are denoted by name, acronym and locus tag (DVU#).

**FIGURE 5:**
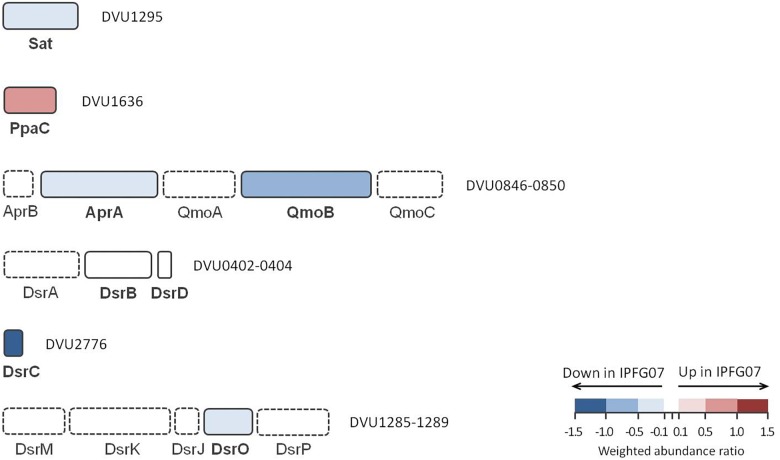
Schematic representation of proteins directly involved in sulfate reduction pathway displayed according to their gene cluster arrangement. The color code of each protein follows the weighted abundance ratio data obtained for the group of 99 proteins that were differentially expressed between the two strains. Proteins not found in this dataset are denoted with dashed boxes.

The proteins directly involved in sulfate respiration during net MSR are most likely to be involved in S isotope fractionation, and their expression profiles are summarized in Figure 5. Protein expression ratios are reported as IPFG07:WT on a log_2_ scale. In IPFG07, two enzymes involved in the activation of sulfate to APS showed opposing expression patterns: sulfate adenylyl transferase (Sat; DVU129) was under-expressed relative to WT (–0.32 ± 0.21) and pyrophosphatase (Ppa; DVU1636) was over-expressed (0.61 ± 0.18). Sat is strictly involved in sulfate reduction, but PpaC is involved in many processes where pyrophosphate degradation is necessary. The APS reductase subunit AprA (DVU0847) showed lower relative expression in the mutant (–0.35 ± 0.21), as did a subunit, QmoB (DVU0849), of its affiliated electron donor complex (–0.52 ± 0.27). The DsrB subunit (DVU0403) of dissimilatory sulfite reductase was slightly (though significantly) more abundant in the mutant (0.07 ± 0.04).

DsrC (DVU2776) was substantially less expressed (−1.13 ± 0.22) in the mutant relative to the WT, as expected given the manipulation of *dsrC* expression in the mutant. From the membrane bound DsrMKJOP complex that reduces the DsrC trisulfide and releases H_2_S, only DsrO (DVU1287) was detected in all replicates. DsrO was less abundant in the mutant (−0.37 ± 0.04) (Figure 5 and Supplementary Figure S5).

### Energy Metabolism Proteins Interacting With DsrC

**FIGURE 6:**
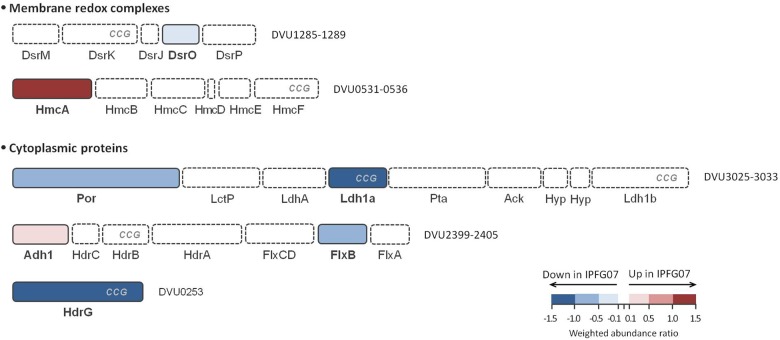
Schematic representation of possible DsrC interacting proteins and their arrangement according to each gene cluster. These proteins belong to HdrB- and HdrD-like proteins, and are identified with CCG code. The color code of each protein follows the weighted abundance ratio data obtained for the group of 99 proteins that were differentially expressed between the two strains. Proteins not found in this set are denoted with dashed boxes. DsrMKJOP is also presented in Figure 5 for reference.

In addition to DsrMKJOP (Figure 5), other enzymes have been postulated to donate electrons to, or otherwise interact with, DsrC (Venceslau et al., 2014). Candidates for this include the Hdr-like membrane complexes TmcABCD and HmcABCDEF. Further possibilities include the soluble proteins involved in lactate (several lactate dehydrogenase subunits) and in ethanol metabolisms (Adh-FlxABCD-HdrABC), as well as other proteins (such as HdrGs). From the FlxABCD-HdrABC complex and its associated alcohol dehydrogenase (Adh1), several subunits were detected in the 276-protein dataset, but only Adh1 (DVU2405, 0.26 ± 0.12) and FlxB (DVU2400, −0.53 ± 0.28) were differentially expressed in the 99 protein dataset (Supplementary Figure S3). Other DsrC-interacting proteins that changed included the large subunit of the high molecular-weight cytochrome complex (HmcA; DVU0536, 1.30 ± 0.25). This protein was the most over expressed protein in the mutant of the *energy production and conservation* category. Finally, HdrG, a heterodisulfide reductase-like subunit of a flavin adenine dinucleotide (FAD) oxidoreductase, was less abundant in the mutant (DVU0253, –1.39 ± 0.05) (Figure 6 and Supplementary Figure S5).

### Carbon Metabolism Proteins Interacting With DsrC

We examined the expression profiles of proteins affiliated with lactate metabolism. Lactate was the carbon source for all growth experiments. The putative Fe-S lactate dehydrogenase, Ldh1a, was less abundant in the mutant than wildtype (DVU3028, –1.52 ± 0.25). Ldh1a belongs to a gene cluster found in the “organic acid oxidation region” (DVU3025 to DVU3033), previously identified in the genomes of *D. vulgaris* and *Desulfo vibrio alaskensis* G20 (Pereira et al., 2007; Wall et al., 2008), and recently named as the *luo* operon (*lactate utilization operon*) (Vita et al., 2015). Another enzyme in the *luo* operon, pyruvate:Fd oxidoreductase known as Por (DVU3025; −0.83 ± 0.23) was also significantly under-expressed. These are all plotted in Figure 6 and Supplementary Figures S3, S5.

### Other Carbon and Energy Metabolism Proteins

Proteins involved in carbon and energy metabolism but less directly involved in sulfate respiration were also identified. Lactate dehydrogenase LldD (DVU2784, −0.45 ± 0.19), was less abundant in IPFG07. Unlike the lactate dehydrogenases discussed above, this enzyme does not belong to the list of putative partners of DsrC (Venceslau et al., 2014; Rabus et al., 2015). Despite indirect involvement in lactate metabolism, several formate dehydrogenases were less abundant in IPFG07: subunits FdhA (DVU2482, –0.36 ± 0.18) and FdhB (DVU2481, −0.53 ± 0.22) of the FdhABC3, and subunit FdhB (DVU0588, –1.33 ± 0.21) In addition, only the [NiFe] periplasmic hydrogenase catalytic subunit HynA (DVU1922, −0.63 ± 0.19) was less abundant in the mutant. We detected one differentially expressed subunit of the Ech membrane-bound hydrogenase, EchD (DVU0431, –0.32 ± 0.06), which was less abundant in IPFG07. From the Na^+^-translocating Rnf complex, RnfC (DVU2792, –0.06 ± 0.04) was slightly down. *D. vulgaris* has an F-type ATP synthase (Ozawa et al., 2000), where subunit AtpD (DVU0774, 0.63 ± 0.39) was more abundant in IPFG07. From the several transmembrane complexes annotated in the genome of *D. vulgaris*, we only detected the QrcB subunit from the quinone reductase complex, more abundant in IPFG07 (DVU0698, 0.75 ± 0.34). This complex receives electrons from periplasmic cytochromes to reduce the membrane-bound quinone pool (Venceslau et al., 2010). Another protein related to energy metabolism, an NADH oxidoreductase (DVU3212, –0.80 ± 0.37) was down in the mutant. This protein is highly conserved among sulfate reducers, and has been reported to reduce AprAB (Chen et al., 1994). These are all plotted in Supplementary Figures S3, S5.

### Other Significantly Changed Proteins

The most under-expressed proteins in IPFG07 are spread among COG functional groups other than energy metabolism (Figure 6). These included under-expression of the leucine biosynthesis proteins LeuA (2-isopropylmalate synthase, DVU2981, −2.75 ± 0.61), and LeuB (3-isopropylmalate dehydrogenase, DVU2985, −0.88 ± 0.13), both of which are encoded in a predicted five-gene operon DVU2981–2985. We also identified strong under-expression of a MTH1175-like domain family protein abbreviated as Mrp (DVU2109, –2.20 ± 0.3). The most over-expressed non-energy metabolism proteins in IPFG07 were: a methyl-accepting chemotaxis protein (DVU0645, 1.77 ± 0.26), a hypothetical protein (DVU0797, 1.41 ± 0.40), as well as a series of ribosomal proteins, RplFLWXU (DVU1318, 0.42 ± 0.20; DVU2927, 0.42 ± 0.10; DVU1305, 0.59 ± 0.10; DVU1314, 0.73 ± 0.10; DVU0927, 1.07 ± 0.30) and RpsACEGL (DVU3150, 0.41 ± 0.28; DVU1309, 0.93 ± 0.10; DVU1320, 0.69 ± 0.30; DVU1299, 0.74 ± 0.20; DVU1298, 1.14 ± 0.10), as well as molecular chaperones GroEL (DVU1976, 0.17 ± 0.10) and GroES (DVU1977, 0.93 ± 0.30). Several proteins related to the inactivation of reactive oxygen species were also found, including superoxide reductase (Sor, DVU3183, –1.68 ± 0.06, c.f. Moura et al., 1990; Sheng et al., 2014), which was one of the most depleted proteins in IPFG07, along with hybrid cluster protein 2, HCP2 (DVU2543, –0.95 ± 0.30), and a rhodanese-like protein (DVU3037, –1.22 ± 0.30). The 99 (of 276) differentially expressed proteins, including those noted above, are identified in Supplementary Figure S3. Most of the 167 proteins detected in all five replicates that were also statistically unchanged between the two strains were not associated with energy metabolism (see Dataframe 02).

## Discussion

In this study we aimed to determine how changes in the availability of DsrC affects sulfur isotopic fractionation, and to what extent that might be mediated by a proteomic response. A key aspect of interpreting these results is the growth in the chemostat. While the wildtype can both grow and reduce sulfate faster than the mutant strain, the turnover time of the chemostat sets the growth rate of both strains to those slower than the maximum for the mutant, and significantly slower for the wildtype. Under these conditions, the csSRR of each strain was the same and invariant (Figure 3). In recent batch experiments these same *D. vulgaris* strains, WT and IPFG07, were cultivated at a higher temperature in nutrient rich medium, and showed small differences in their sulfur isotopic fractionation and growth rates, within the larger analytical uncertainties of that study (Table 1), although csSRR was not quantified (Leavitt et al., 2016). Herein both strains grew more slowly in batch due to the lower temperature and less rich medium, and slower still in the chemostats. A comparison of the prior study and the batch and chemostat work here are presented in Table 1.

**Table 1:** Comparison of key growth parameters and isotopic fractionations in batch or chemostats.

Strain	^∧^μ_batch_ (h^−1^)^∧^	^#^μ_batch_ (h^−1^)^#^	^∧^μ_chemostat_ (h^−1^)^∧^	^∧^csSRR_chemostat_ (*f*mol/cell/day)^∧^	^34^ε_batch_ (‰)^#^	^34^ε_chemostat_ (‰)^∧^
WT	0.24	0.15	0.03	6.2 ± 1.4	13.1 ± 1.3	17.3 ± 0.5
IPFG07	0.05	0.07	0.03	6.7 ± 1.0	15.3 ± 1.3	12.6 ± 0.5

*Symbols (∧) indicate this study, while (#) refer to Leavitt et al. (2016). Batch and chemostat experiments in this study were conducted at 30.5 ± 0.1°C, whereas Leavitt et al. (2016) worked at 37°C.*

These results can be interpreted by comparing the rates and fractionations. This study shows that at equal growth rates and csSRR, the wildtype strain produces a larger fractionation – an intrinsic difference between the strains. There is a well-documented relationship between relationship between fractionation and csSRR (Kaplan and Rittenberg, 1964; Chambers et al., 1975; Sim et al., 2011a,b; Leavitt et al., 2013). Thus, at faster rates we would expect both IPFG07 and the WT to have smaller fractionations than in this study. However, that is not what has been observed in the fast batch growth experiments. The breakdown of this pattern could be a consequence of responses in either strain to changes in conditions over the course of batch growth. In either case, the decoupling of rate from sulfur isotopic fractionation suggests that the metabolic processes producing the fractionation are not consistent between experiments.

Within this experiment, we seek to explain the observation that IFPG07 shows a smaller isotopic fractionation than the WT, which must relate to the fluxes and fractionations of sulfur through its metabolic pathway – which is informed by proteomics. Due to its limited DsrC pool, IPFG07 is operating much closer to its maximum possible csSRR than WT in these chemostats. Under the rate constraint imposed by the chemostat conditions, IPFG07 grew at 83% of the maximum batch-culture observed doubling time (20.8 vs. 25 h), whereas WT grew at only 17% of its maximum batch-culture doubling time (4.2 vs. 25 h). Here we are comparing to the batch-growth rates under the same medium and temperature conditions (30°C), which are different from those in Leavitt et al. (2016) (37°C). The IPFG07 mutant appears to have a lower inherent capacity for sulfate reduction – a consequence of the under-expression of DsrC. Because a standing cytoplasmic pool of reduced DsrC is required to efficiently remove zero-valent sulfur from the catalytic site of DsrAB, when DsrC is under-expressed, this limits the cells inherent capacity for sulfate reduction, particularly through catalysis of the downstream part of the pathway. The smaller fractionation by IPFG07 may be a consequence of: (i) the intracellular accumulation of intermediates upstream of DsrC, such as sulfite, allows for the expression of all steps up to and including, DsrC; (ii) differences in the flux of sulfur through the organism due to the production of alternate sulfur-bearing phases such as thionates (unlikely in this case); and/or (iii) the additional production of H_2_S from pathway that does not involve DsrC, such as the direct reduction of sulfite to H_2_S by DsrAB (Santos et al., 2015). We cannot distinguish between these hypotheses, though seek to do so in future works now that the analytical tools exist to aid such attempts (Sim et al., 2017).

The proteomic data are consistent with a cellular-level response to low DsrC. Protein expression profiles of other parts of the sulfate reduction pathway showing mirrored the under-expression of DsrC. For example, Sat, AprA, and QmoB were all under-expressed in IPFG07. These enzymes are all related to the cellular machinery that delivers sulfur to DsrC. Similarly, there is evidence for decreased electron delivery generally, through under-expression of Ldh1a which processes the electron donor, lactate (Vita et al., 2015). There is evidence for decreased electron delivery to DsrC through under-expression of DsrO.

These results are consistent with a cellular response to low DsrC that optimizes relative protein expression levels. Not all proteins in the sulfate reduction pathway showed this response – in particular, DsrAB subunits were minimally affected, which agrees with the lack of co-regulation observed between the *dsrC* and *dsrAB* genes in an early report (Karkhoff-Schweizer et al., 1993). However, by attenuating delivery of sulfur and electrons to Dsr enzymes, the cells may gain advantage by preventing bottlenecks or catalysis of adverse reactions that could occur in the absence of sufficient DsrC (e.g., production of thionates at DsrAB). Acclimation of cells need not occur at every enzyme, rather is only necessary in a sufficient number of subunits where reaction catalysis is inhibited. For example, APS reduction can be inhibited by reducing the availability of the catalytic subunit of APS reductase (AprA), even if AprB is unperturbed. Previous analyses of *D. vulgaris* have shown that the abundance of transcripts encoding AprA and AprB adjust to growth conditions, but not in identical fashion (Haveman et al., 2003). Similarly, under-expression of the ferredoxin-like electron-transferring subunit DsrO (Figure 5) may be sufficient to prevent the DsrMKJOP complex from catalyzing off-target reductions, when insufficient electron acceptors, in the form of oxidized DsrC, are available.

Other protein data also suggest that the cellular response to the DsrC shortage is to restrict electron flow. The Flx–Hdr complex has been predicted to donate electrons to DsrC (Ramos et al., 2015), and FlxB was less abundant in IPFG07 (Figure 6). In contrast, the alcohol dehydrogenase Adh1 was up-regulated, and is co-localized with the Flx–Hdr complex on the chromosome (Figure 6 and Supplementary Figure S5). This enzyme is involved in ethanol metabolism, but is highly expressed and plays a role in lactate metabolism (Haveman et al., 2003). In addition, LeuA catalyzes the first step of leucine synthesis with the conversion of 2-ketoisovalerate and acetyl-CoA to 2-isopropylmalate and CoA, respectively. Due to use of acetyl-CoA in this reaction, this biosynthesis is interlinked with pyruvate metabolism. This may suggest that by lowering the expression of LeuA, less acetyl-CoA is being deflected to leucine biosynthesis. Additionally, the alanine biosynthesis route also relies directly on pyruvate for its synthesis. In this case, alanine dehydrogenase, Ald (DVU0571, −0.98 ± 0.17), that is responsible for the catalysis of pyruvate to L-alanine, is among the less abundant proteins in the mutant. The response of these two amino acid metabolism routes may suggest that both are working as a compensatory mechanism to favor the electron flow for pyruvate oxidation, due to the decrease in abundance of Ldh1a. As such, the down-regulation of this gene cluster is consistent with the suggestion that Ldh may work as a physiological partner of DsrC when oxidizing lactate coupled to sulfate respiration (Venceslau et al., 2014). This is further consistent with the CCG-domain observed in Ldh1a, common to catalytically active subunits of the heterodisulfide reductase in methanogens (HdrD/HdrB) and others in sulfate reducers (DsrK) (Venceslau et al., 2014). Last, HdrG was also under-expressed in IPFG07 (Figure 6), consistent with the hypothesis that HdrG is a partner of DsrC in the electron transfer pathways from NAD(P)H to DsrC trisulfide (Venceslau et al., 2014).

Few proteins not clearly associated with energy metabolism were significantly changed between strains. This is consistent with a cellular-level response to DsrC under expression, which is primarily central to energy metabolism. Overall the relative activities and associated abundance of the electron donating and electron accepting machinery are responsible for the flux of sulfur and energy through the catabolic arm of the MSR. It is consistent with the hypothesis that the magnitude of S isotopic fractionation responds to the relative fluxes of terminal electron acceptor (sulfate) and primary electron donor (lactate), constrained by overall utilization of each strains sulfate respiration capacity, as dictated by protein availabilities and activities.

## Conclusion

Together, these isotopic and proteomic observations suggest that in *D. vulgaris*, genetically enforced suppression of DsrC expression results in decreased metabolic capacity for sulfate reduction and a smaller expressed sulfur isotopic fractionation. Interestingly, the forced under-expression of DsrC in the mutant strain resulted in perturbations to protein expression both within and beyond the central sulfate reduction pathway, consistent with throttling of the fluxes of sulfur and electrons for sulfate reduction. This work provides the first experimental and quantitative test of changes in the abundance of the MSR machinery (proteins) carrying out S isotope fractionation. These observations show that protein expression indeed matters for net S isotope fractionation. Metabolic models that predict fractionation (c.f. Wing and Halevy, 2014; Bradley et al., 2016; Wenk et al., 2017) need to incorporate the most up-to-date understanding of the MSR network architecture (Santos et al., 2015) and include effects catalyzed by differential regulation, and ultimately protein expression.

DsrC is less abundant in IPFG07 than in the WT, resulting in a smaller fractionation at the same csSRR. This is broadly consistent with recent observations of fractionation as functionally dependent on predicted enzyme abundances (Pellerin et al., 2018). Together this indicates that the magnitude of fractionation responds to the degree of utilization by a cell (or population) of its inherent sulfate reduction capacity. Casting these observations to natural populations, we predict that when comparing localities or discrete time units from within a locality – where the observed sulfur isotope fractionation changes – this shift may indicate changes in protein abundance within or between strains, in response to factors not directly associated with sulfate reduction. To further understand how culture-based observations of stable isotope fractionation play out in nature, we encourage this approach be applied to natural populations over a range of terminal oxidant (sulfate), reductant and/or nutrient availabilities.

## Author Contributions

WL and AB designed the project. WL and SV designed and conducted the experiments. WL conducted S isotope analyses. WL and DS conducted the geochemical analyses. JW performed the proteomic analyses. JW, SV, IP, AB, and WL analyzed the proteomic output. All authors contributed to the manuscript writing.

## Conflict of Interest Statement

The authors declare that the research was conducted in the absence of any commercial or financial relationships that could be construed as a potential conflict of interest.

## Footnotes

1 ftp://massive.ucsd.edu/MSV000083165

2 https://figshare.com/s/e98485f2bf4e2f6817f9

3 https://github.com/WaldbauerLab/mfap-downstreamprocessing

4 http://www.microbesonline.org/

5 http://img.jgi.doe.gov/

6 https://figshare.com/s/e98485f2bf4e2f6817f9

## Supplementary Material

The Supplementary Material for this article can be found online at: https://www.frontiersin.org/articles/10.3389/fmicb.2019.00658/full#supplementary-material and https://figshare.com/s/e98485f2bf4e2f6817f9.
