# Supplementary material for: Proteomic and Isotopic Response of Desulfovibrio vulgaris to DsrC Perturbation
Source: Front Microbiol. 2019 Apr 11;10:658. doi: 10.3389/fmicb.2019.00658 (PMC6470260; doi:10.3389/fmicb.2019.00658)
Supplement: Supplementary file 1 [file Data_Sheet_1.PDF]

Proteomic and isotopic response of *Desulfovibrio vulgaris* to DsrC perturbation

SUPPLEMENTAL FIGURES S1 thru S6

Figure S1

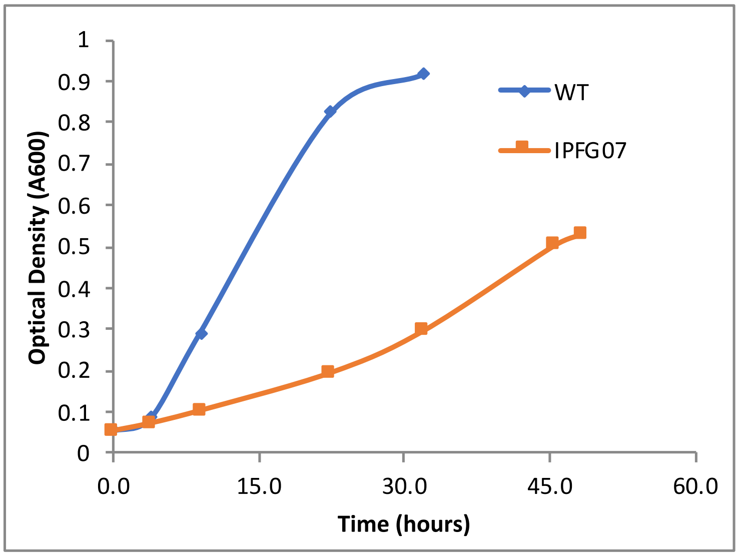

Figure S1. Batch growth experiments under the same medium and temperature conditions as in the chemostats.

Figure S2

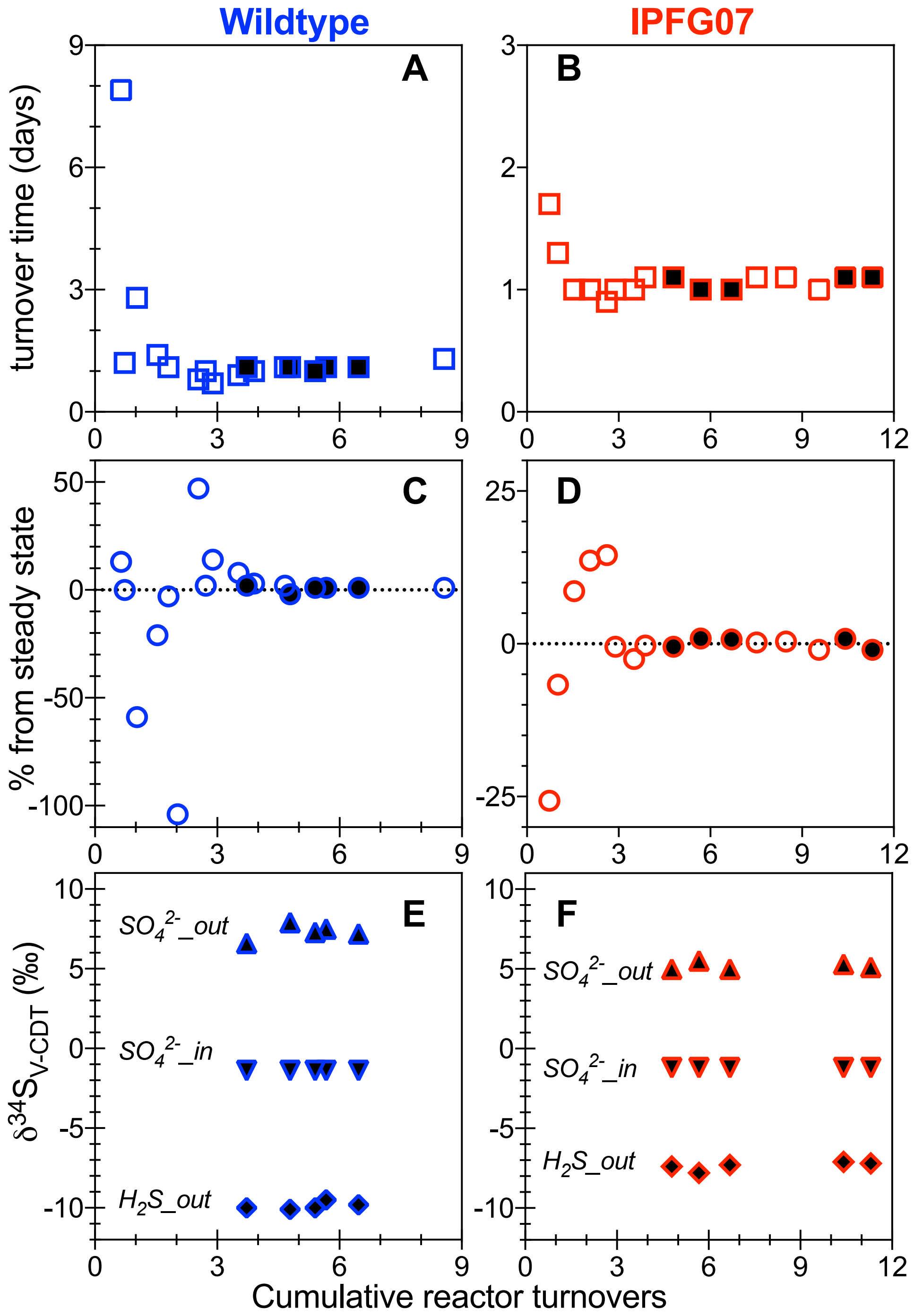

Figure S2. Chemostat data for WT (A,C,E) and IPFG07 mutant (B,D,F). Panel (A, B) Calculated turnover times, (C, D) departure from steady-state; (E, F) measured stable S isotopic compositions. The filled symbols represent the five turnovers (time points) at which proteomic and stable S isotope samples were collected.

Figure S3.

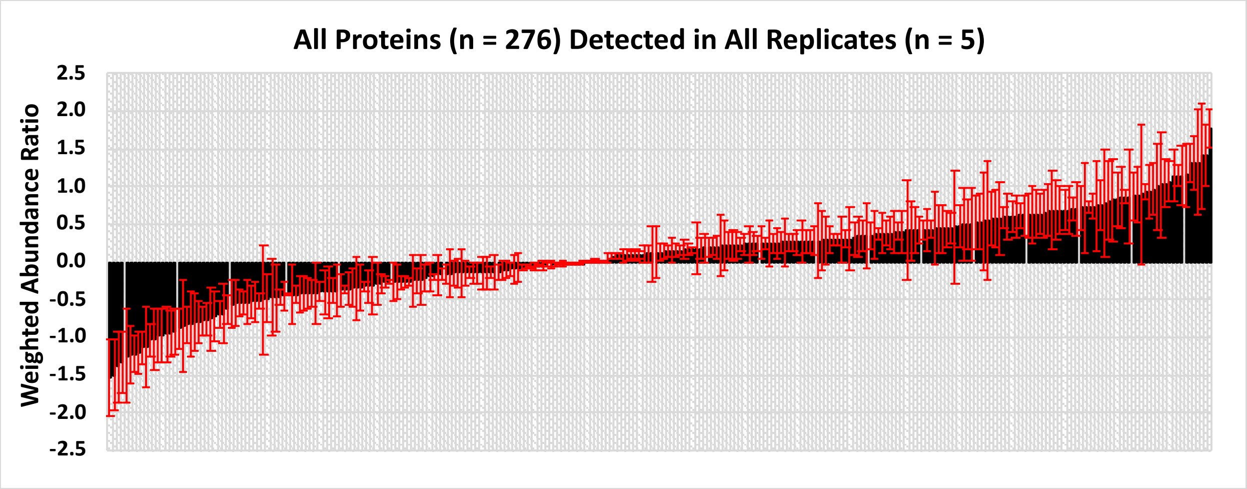

Figure S3. Skyline plot of the Weighted Abundance Ratio's (black bars) and Weighted Ratio Error (red bars) for all 276 proteins detected in all five replicates. This includes both those significantly and insignificantly changes between WT and IPFG07. See Dataframe02 for values and identities. Negative values are less abundance in mutant relative to wildtype.

Figure S4.

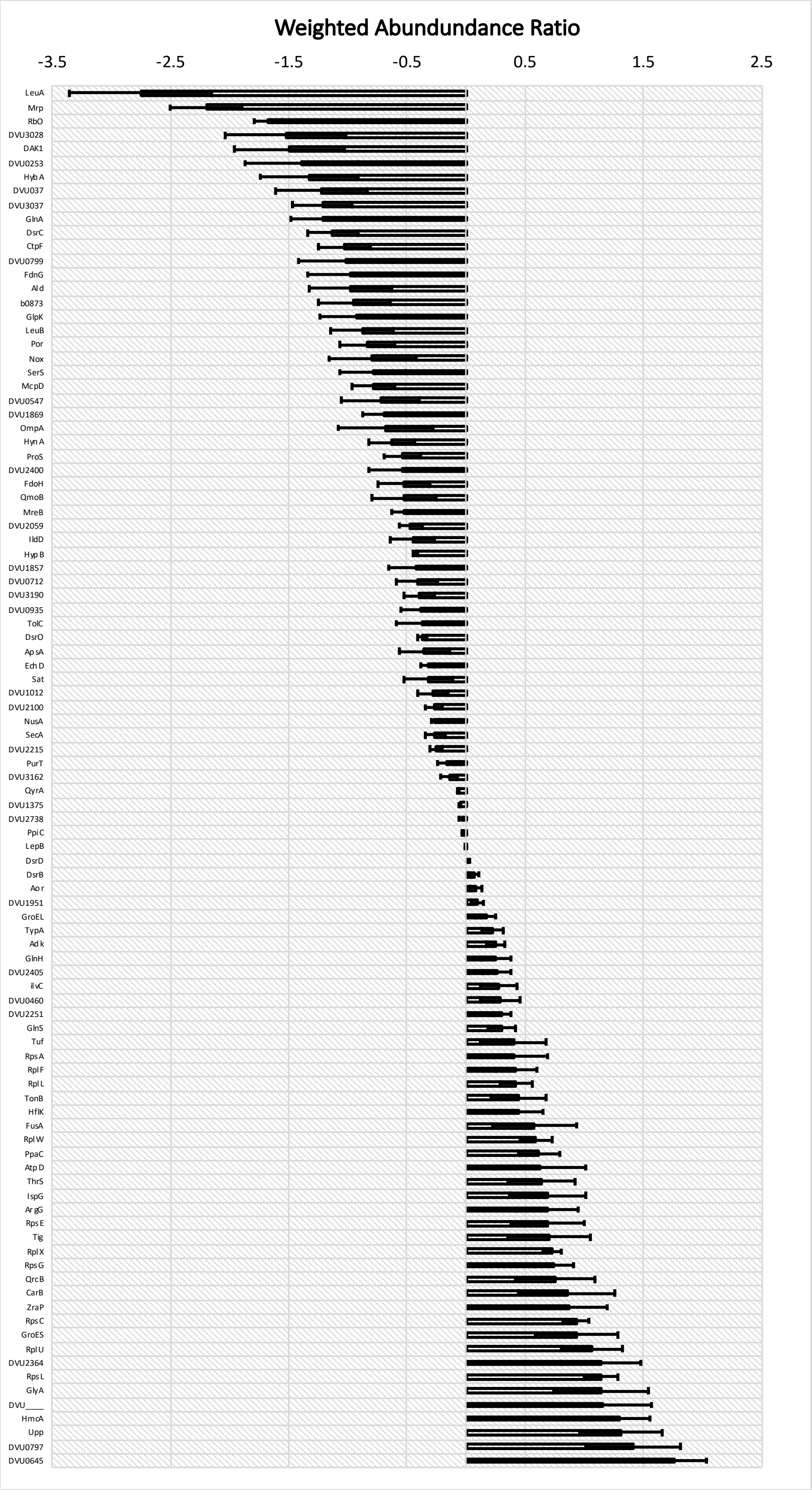

Figure S4. Weighted abundance ratios and weighted ratio errors for all 99 proteins detected in all 5-replicates that were statistically differentially expressed. For reference, negative values are less abundant in mutant relative to wildtype, as in all other plots.

Figure S5.

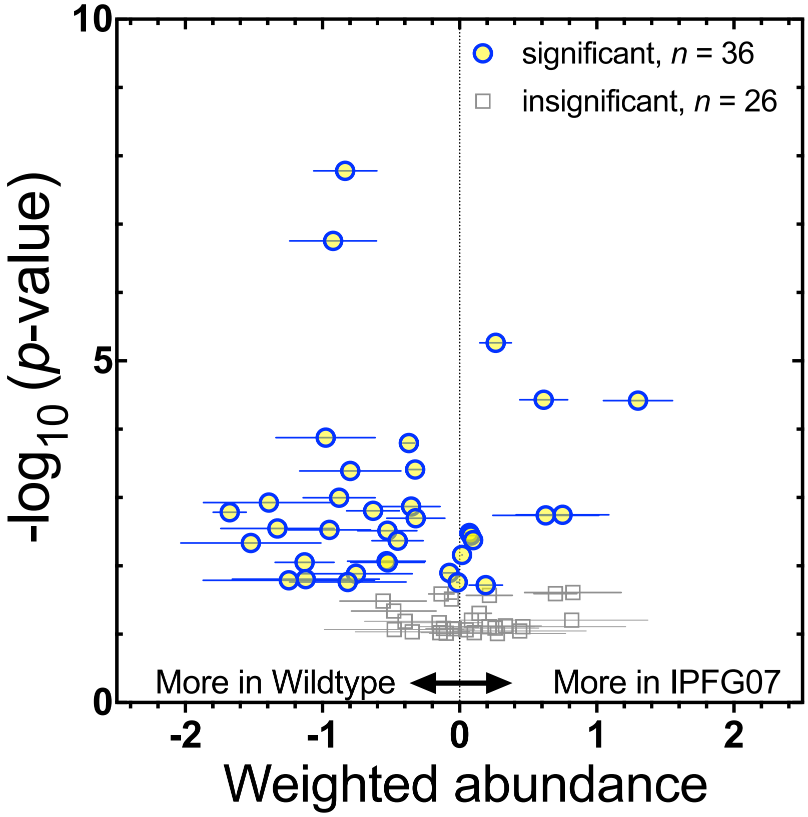

Figure S5. Differentially expressed energy metabolism proteins in all five replicates on a volcano plot. COG categories were used to identify the *Energy Metabolism* group. From 210 known Energy Metabolism COG identified genes in *D. vulgaris* Hildenborough, 62 were detected in all five replicates in both strains. (X-axis) Weighted abundance versus the (Y-axis) *p*-value for proteins significantly (blue circles) or not significantly (grey squares) different between the strains. Positive values indicate more of a protein in the IPFG07 mutant relative to WT, negative values indicate less. Error estimates are weighted ratio error (*see* Methods for calculations).

Figure S6.

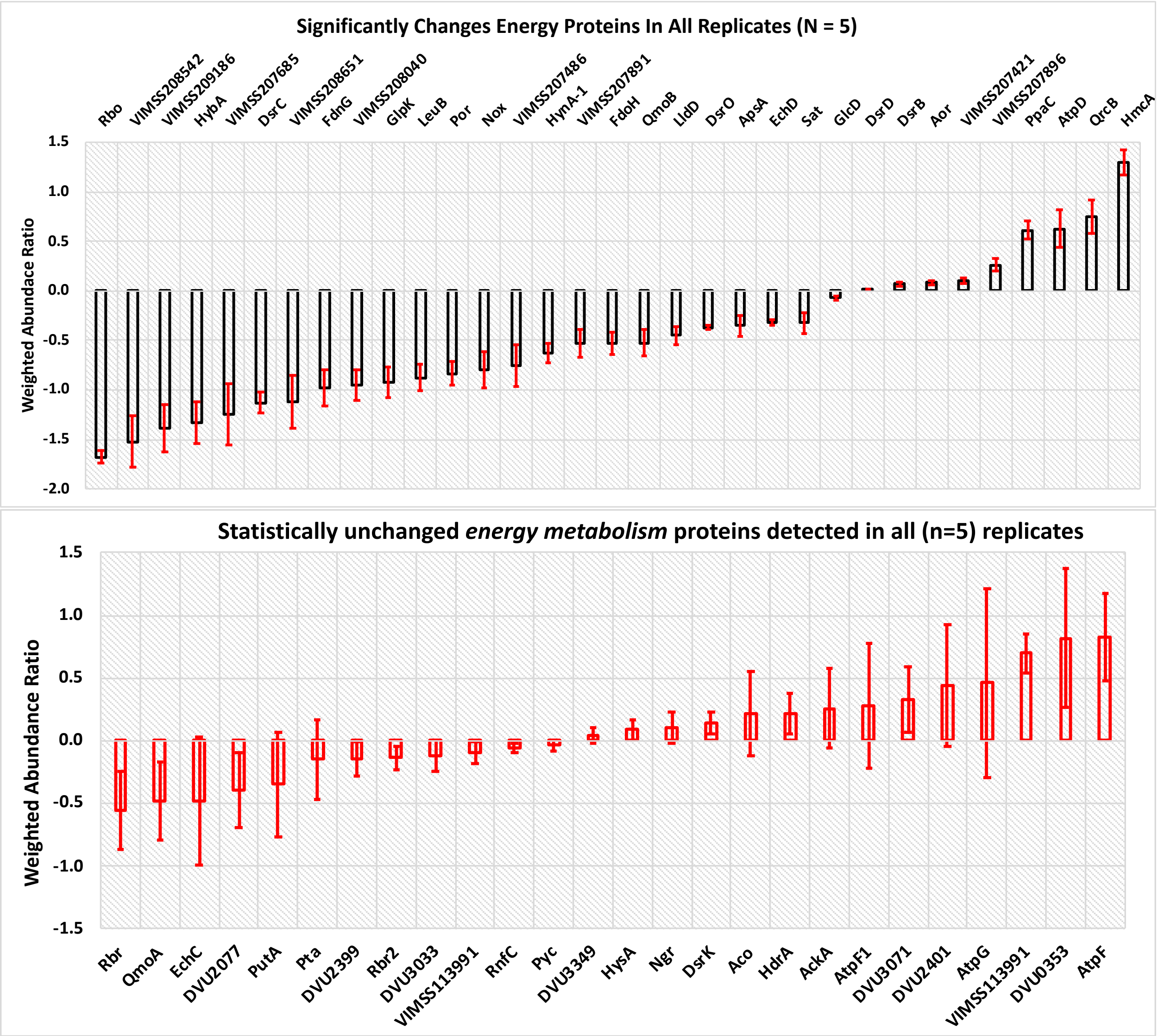

Figure S6. (top) The significantly changes energy metabolism proteins between IPFG07 and WT. (bottom) the unchanged energy metabolism proteins. These are from the 276 proteins detected in all five experimental replicates.
